# Improving auditory alarm sensitivity during simulated aeronautical decision-making: the effect of transcranial direct current stimulation combined with computerized working memory training

**DOI:** 10.1186/s41235-025-00620-x

**Published:** 2025-03-07

**Authors:** Rongjuan Zhu, Xiaoliang Ma, Ziyu Wang, Qi Hui, Xuqun You

**Affiliations:** 1 College of Management, Xi’an University of Science and Technology, Xi’an, 710054 China; 2 GEOVIS Earth Technology Co., Ltd., Hefei, 230088 China; 3 Institute of Social Psychology, School of Humanities and Social Sciences, Xi’an Jiaotong University, Xi’an, 710049 China; 4 Key Laboratory for Behavior and Cognitive Neuroscience of Shaanxi Province, School of Psychology, Shaanxi Normal University, Xi’an, 710062 China; 5 School of Psychology, Shaanxi Normal University, Yanta, Xi’an, 710062 China

**Keywords:** Auditory alarm deafness, Inattentional deafness, Working memory training, DLPFC

## Abstract

Auditory alarm deafness is a failure to notice a salient auditory signal in a high-load context, which is one of the major causes of flight accidents. Therefore, it is of great practical significance for aviation safety to explore ways to avoid auditory alarm deafness under a high-load scenario. One potential reason for its occurrence could be the fact that cognitive resources are limited. Working memory (WM) capacity is important for the availability of cognitive resources. The present study investigated the effects of different types of WM ability and transcranial direct current stimulation (tDCS) combined with WM training on auditory alarm sensitivity in a simulated high-load aeronautical decision-making task in two experiments, with participants who were not trained pilots. The results showed that different types of WM storage capacity did not predict auditory alarm deafness. However, individuals with high executive function of WM were more sensitive to the auditory alarm than those with low executive function. During WM training, tDCS over the right dorsolateral prefrontal cortex not only improved WM executive function but also improved auditory alarm sensitivity under high-load conditions. These findings suggest that the storage and executive function of WM have different roles in auditory alarm sensitivity. WM training based on brain stimulation technology can provide empirical evidence for the enhancement of auditory alarm alertness and cognitive function in the human–machine context.

## Introduction

In safety production, an auditory alarm can provide critical emergency signals for operators. However, many studies have shown that high-load visual tasks prevent the processing of task-irrelevant auditory stimuli, i.e., they cause inattentional deafness (Dehais et al., 2014; Macdonald & Lavie, 2011; Murphy & Greene, 2015). Inattentional deafness can pose a significant safety risk, especially in aviation. The analysis of air accident reports has shown that many flight accidents are due to inattentional deafness, also known as auditory alarm deafness (Bliss, 2003; Mumaw, 2017). Therefore, there is great practical significance for aviation safety to explore the mechanism of alarm deafness and how to avoid it.

Load theory indicates that high-load tasks consume the most cognitive resources, leaving little or none available for processing task-irrelevant auditory stimuli (Lavie, 1995). Some studies conducted in both simulated (Dehais et al., 2016; Massé et al., 2022; Somon et al., 2022; Zhu et al., 2023) and real-world flight conditions (Dehais et al., 2018) have found that limited central cognitive resources can explain the phenomenon of auditory alarm deafness. People with more cognitive resources tend to exhibit better attention abilities, both divided and selective (Colflesh & Conway, 2007), and thus should be more likely to detect a critical auditory alarm in a high-load context. Working memory (WM), often described as the cognitive system responsible for temporary storage and manipulation of information, serves as the most intuitive reflection of cognitive resources (Kane & Engle, 2002). It is a fundamental cognitive ability that predicts performance on a wide range of advanced cognitive skills (Klingberg, 2010), including auditory selective attention (Dalton et al., 2009). This suggests a potential link between WM and auditory alarm deafness.

The relationship between WM and auditory alarm deafness currently involves two different theoretical hypotheses: the single-route theory and the dual-route theory (Lewis, 2017; Zhu & You, 2022). The single-route theory indicates that individuals with high WM capacity are more likely to detect a critical auditory alarm than those with low WM capacity. This is because individuals with high WM have enough cognitive resources to process task-irrelevant stimuli. Contrary to this single-route model, Kreitz et al. (2016) found that WM capacity, as measured by performance on spatial and verbal 2-back tasks as well as the automated operation span task, does not predict inattentional deafness. Dehais et al. (2019) similarly found no association between WM capacity and auditory alarm deafness. Given these inconclusive findings, the dual-route model suggests a possible inverted U-shaped functional relationship between WM capacity and detection rate (Hannon & Richards, 2010). Specifically, this model posits that individuals with low WM capacity will fail to notice critical stimuli in high-load contexts because they have fewer cognitive resources to process task-irrelevant stimuli, while individuals with high WM capacity do not detect the critical stimuli because they over-inhibit attention to task-irrelevant stimuli. Lewis (2017) found that individuals with both high and low WM capacity failed to notice a critical auditory alarm in a high-load cross-modality condition, consistent with the dual-route model. Taken together, these findings suggest that it is still unclear whether the relationship between WM capacity and auditory alarm deafness in high-load aviation scenarios conforms to the single-route or the dual-route model.

Previous research on the relationship between WM and auditory alarm deafness is sparse and presents considerable inconsistencies in findings (Dehais et al., 2019; Kreitz et al., 2016; Lewis, 2017). These discrepancies may stem from the tendency of existing studies to conceptualize WM as a monolithic construct, thereby neglecting the distinct components and functions that constitute it. WM is a very complex system consisting of the phonological loop, visual-spatial sketch pad, and central executive subsystems (Baddeley & Hitch, 1974). Engle’s executive attention theory builds on this complexity by suggesting that WM’s efficiency heavily relies on attention and executive control to manage its limited capacity and update information (Engle, 2002). Updating, inhibition, and task switching are important components of the executive function of WM, and the updating function is more closely related to other higher cognitive abilities than are inhibition and task-switching (Chen & Li, 2007; Friedman et al., 2006). A wealth of research indicates that distinct WM subcomponents possess distinct functionalities and characteristics (Hartley et al., 2016; Kofler et al., 2018; Linck et al., 2014). Moreover, Kim et al. (2005) indicated that various components of WM exert differential effects on selective auditory attention. Hence, the impact of different modules of WM on selective attention may vary. It is unclear whether specific aspects of WM storage and the executive function of WM have dissociable effects on auditory alarm deafness.

WM training not only enhances WM capacity but also improves cognitive abilities such as attention and learning (Jones et al., 2020; Lilienthal et al., 2013; Pappa et al., 2020). Training combined with transcranial direct current stimulation (tDCS) has been found to be more effective in cognitive enhancement than a single training method (Fridriksson et al., 2011). tDCS can alter cortical excitability in a specific area of the brain by applying a weak electric current through electrodes placed on the scalp. The directions of this change in excitability depends on the polarity of the electrodes (Fregni et al., 2005). Anodal stimulation induces depolarization of the neuronal resting membrane potential, leading to increased neuronal excitability. Conversely, cathodal stimulation causes hyperpolarization of the resting membrane potential, resulting in reduced neuronal excitability (Brunoni et al., 2013). As an effective tool for exploring the causal relationships between brain regions and their corresponding cognitive functions, tDCS has been widely used in cognitive fields including the study of WM (Berryhill, 2017) and attention abilities (Mannarelli et al., 2019). These studies suggest that leveraging tDCS combined with WM training to enhance brain function may offer potential for improving auditory alarm sensitivity in high-load scenarios.

The WM training effect transfers more easily to other cognitive abilities when there is an overlap in the brain regions associated with WM and other higher cognitive skills (Dahlin et al., 2008; Owen et al., 2005). Studies on the neural mechanisms of auditory alarm deafness have shown that an ignored auditory alarm will activate the frontal and temporal cortical regions associated with visual-auditory WM (Dehais et al., 2018; Durantin et al., 2017). The right dorsolateral prefrontal cortex (DLPFC) is a critical region within the frontal cortex that plays a central role in higher-order cognitive functions, including inhibitory control, attention, and WM (Friedman & Robbins, 2022). Previous research has demonstrated that the DLPFC is integral to managing attentional resources, which is crucial for mitigating inattentional deafness. Specifically, the DLPFC is involved in integrating the dorsal attention network (DAN) and the ventral attention network (VAN) during task-switching and attention shifts, as evidenced by increased gamma-band activity in this region (Tamber-Rosenau et al., 2018). This integration facilitates the selective enhancement of task-relevant processes and the suppression of task-irrelevant processes. Given its role in inhibiting irrelevant stimuli, stimulating the right DLPFC might enhance the processing of relevant alarms. However, despite these potential benefits, it remains unclear whether tDCS combined with training specifically targeting the right DLPFC can more effectively improve auditory alarm sensitivity in high-load aviation scenarios.

The current study aims to investigate whether WM capacity plays an important role in auditory alarm deafness during high-load aeronautical decision-making tasks and how to improve auditory alarm sensitivity from the perspective of WM. For that purpose, we adopted a high-load aeronautical decision-making task from Zhu et al. (2022) in Experiment 1 to explore, using signal detection analysis, whether visual-spatial WM, verbal WM storage capacity, and the executive function of WM have different effects on auditory alarm detection. Moreover, given the dual-task and cross-modality characteristics of visual-auditory alarm detection tasks, the visual-auditory n-back task was chosen here; it measures the updating function, the main task of the executive function of WM. We hypothesize that, according to the single-route model, there will be a linear relationship between WM capacity and auditory alarm detection performance, with higher WM capacity being associated with better detection performance. Conversely, based on the dual-route model, we hypothesize that the relationship between WM and auditory alarm detection performance may instead exhibit a U-shaped curve, indicating that both low and high WM capacities might be associated with poorer detection performance, while moderate WM capacity is expected to be associated with optimal performance. Experiment 2 aimed to explore ways to avoid or reduce auditory alarm deafness — in other words, to explore effective ways to improve auditory alarm sensitivity in high-load aeronautical decision-making contexts. We hypothesized that tDCS combined with WM training would elicit greater transfer effects on the auditory alarm detection task than either sham tDCS with WM training or tDCS alone. By adopting signal detection analysis with four key metrics—hit rate, false alarm rate, discrimination index, and response bias – we aim to comprehensively assess participants’ sensitivity to auditory alarms and their decision-making biases, consistent with methodologies in previous studies (Raveh & Lavie, 2015; Zhu et al., 2022).

## Experiment 1

### Materials and methods

#### Participants

In this study, 90 healthy undergraduate students were recruited from Shaanxi Normal University (mean age = 19.53 ± 2.41 years, 47 women). However, six participants withdrew because of low accuracy on the mathematical equations and symmetry judgment portion of complex WM span tasks. Ultimately 84 healthy participants (mean age = 19.31 ± 2.18 years, 43 women) were included in the final sample. They were right-handed and had normal or corrected-to-normal vision, normal hearing, and were able to detect the auditory stimulus used in the experiment upon hearing the sound examples. They were unaware of the purpose of the experiment. Prior to the study, written informed consent was obtained from all participants in accordance with the requirements of the ethics committee at Shaanxi Normal University (HR2020-10-001).

#### Stimuli and tasks

##### Auditory alarm detection task during aeronautical decision-making

**Fig. 1:**
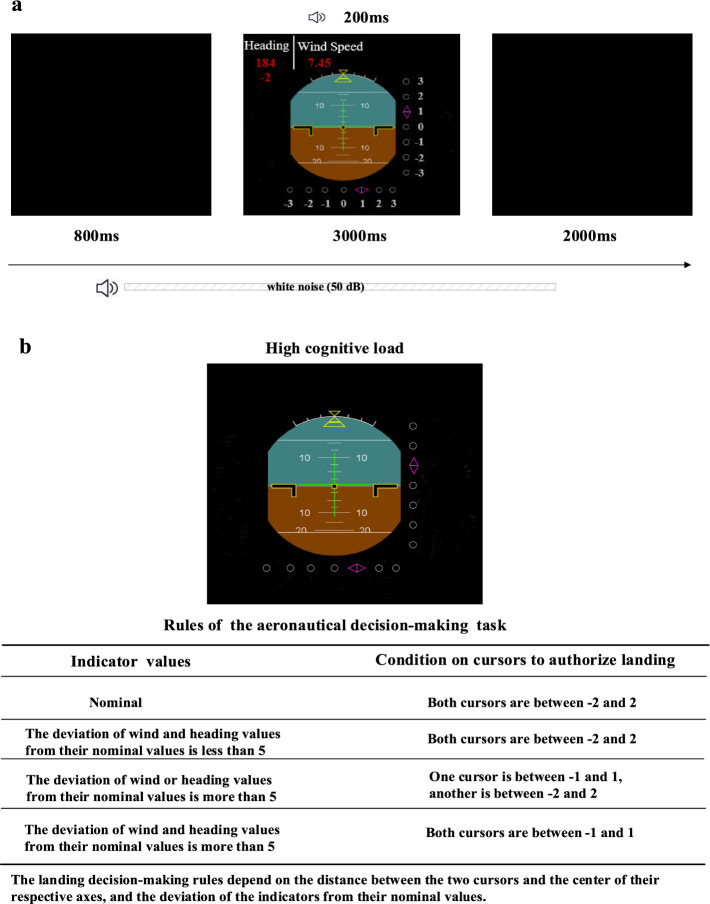
A schematic of the auditory alarm detection task during aeronautical decision-making scenario. **a** Experimental procedures: An example of one trial showing the sequence of events and timing. **b** The rules for the high-load aeronautical decision-making task

In Experiment 1, the participants were seated 60 cm away from a monitor and were presented with a series of stimuli (Fig. 1a). Each trial consisted of a fixation point (subtending a 2.6° × 2.6° visual angle) displayed for 800 ms, followed by a Primary Flight Display (PFD) for 3000 ms. The PFD, based on the one used in Airbus 320 cockpits, included various indicators such as heading, the magnetic declination, and the wind speed displayed in the upper left corner, with the magnetic declination shown below the heading. The Instrument Landing System, represented by two cursors (localizer and glide) providing lateral and vertical guidance for the aircraft’s trajectory relative to the runway, was displayed as two rhombi below and to the right of the artificial horizon (Giraudet et al., 2015). White noise at 50 dB (SPL) was played continuously during the task and an auditory alarm (1250 Hz) at 78 dB (SPL) lasting 200 ms was randomly played between 200 ms after the start and 1000 ms before the end of the PFD stimulus. The participants were instructed to pretend they were flying a plane that had reached the decision altitude, at which point the pilot must decide whether to abort the landing or not, and were to follow the rules for high-load landing decision-making as outlined in Zhu et al. (2022), using the cursors and indicators on the PFD (Fig. 1b). Upon presentation of a question mark on the display, participants responded by pressing either the letter “F” of a standard keyboard for landing or the letter “J” for no landing, and pressing “V” on the keyboard if they heard an auditory alarm. Before the formal experiment began, all participants went through a practice session to become familiar with the requirements. To prevent practice effects, the auditory stimuli in the practice session differed from those in the formal experiment, with the practice alarm set at a frequency of 1500 Hz. Participants completed 20 trials during the practice session and 200 trials in the experimental session. The auditory alarm was presented in only 20% of the trials.

##### WM tasks

Both the complex verbal and spatial operation span task and the dual 2-back task were measured using a WM test application developed in our laboratory. This application comprises three core modules: the main test terminal used by the experimenter, the tested terminal used by the experimental participants, and the cloud computing access module. Its functions include training and testing on different WM tasks. The software development environment of the system is Android Studio 4.0, which is officially released by Google. The application is compatible with Android 7.0 and above and can run on various sizes of Android tablets with a resolution of 1200 × 1920. A Huawei 10.4-inch tablet was used in this experiment.

**Fig. 2:**
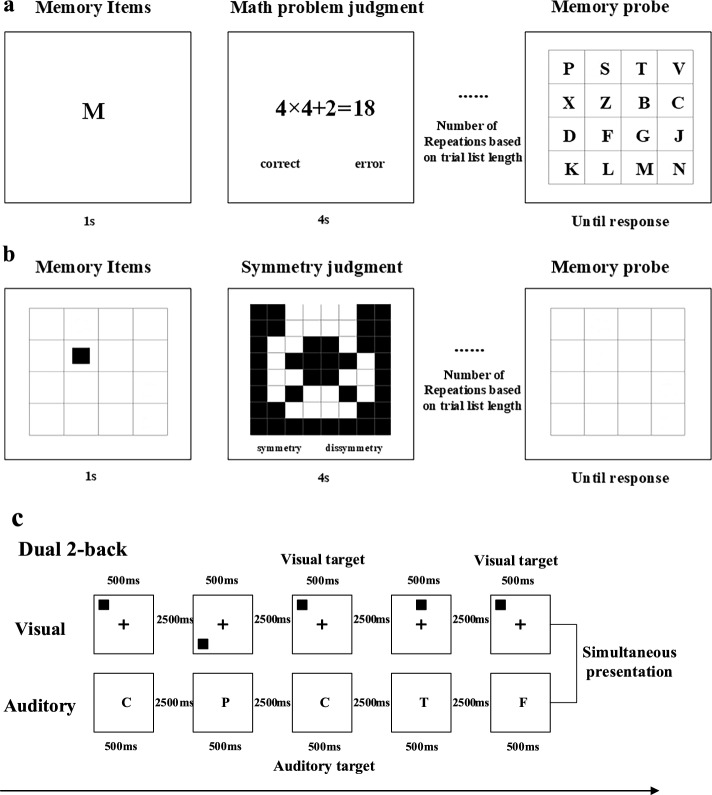
A schematic of each WM task measure. **a** Complex verbal operation span task. **b** Complex spatial WM span task. **c** Dual 2-back task

*Complex Verbal Operation Span Task* The complex verbal operation span task required participants to remember a sequence of letters while also completing a distracting processing task. In each trial, a letter was presented for 1 s in the center of the screen, followed by 4 s of a math problem judgment task. Each to-be-remembered letter was preceded by 4 s of repeated, participant-paced, math problem decision tasks. Finally, a 4 × 4 alphabet was presented and participants were required to recall the letter sequence in the correct order (Fig. 2a).

*Complex Spatial WM Span Task* In the complex spatial WM span task, participants were required to remember the position of a square presented in a sequence of different square locations while completing an interleaved symmetry judgment task. In each trial, a 4 × 4 grid was presented for 1 s in the center of the screen. A small black square appeared within the grid at random, and participants were required to remember the location of the small square. Then, an interleaved symmetry figure was presented for 4 s and participants were required to determine whether the figure was symmetrical. The position of the square and the symmetry judgment were presented alternately, with the number of repetitions based on the trial list length. Finally, the 4 × 4 grid was presented again and participants were asked to recall the position of the black square in the sequence (Fig. 2b).

*Dual 2-back Task* In this task, participants were presented with two series of stimuli simultaneously at a rate of 3 s per stimulus (Fig. 2c). One series consisted of single letters presented through headphones, while the other consisted of individual spatial locations marked with a black square on the screen. Participants were instructed to determine whether the position of the square was the same as the one presented n (*n* = 2) items before in the sequence and whether the letter presented by the auditory modality matched the one presented n (*n* = 2) items before in the sequence. They made their responses by touching the “Visual” button on the tablet’s screen for visual targets, the “Auditory” button for auditory targets, and the “Visual-Auditory” button to indicate a simultaneous match of both visual and auditory targets.

### Data analysis

Firstly, we calculate the WM performance in the complex verbal and spatial WM span task, as well as the dual 2-back task. The WM span task can be scored in several different ways. For the current experiment, we used the partial credit load scoring (PCLS) method described by Conway et al. (2005), in which the score is the sum of all correctly recalled items divided by the total number of items. Accuracy on the dual 2-back task was measured using the d-prime, which reflects the participant’s ability to distinguish targets from non-targets (Gómez et al., 2024; Nikolin et al., 2018). The d-prime was calculated using the formula: d-prime = Z_hit_ – Z_false alarm_ (Haatveit et al., 2010). In addition, we also analyzed the average accuracy and response time of aeronautical decision-making performance. Secondly, we analyzed auditory detection performance using key metrics: hit rate, false alarm rate, discrimination index, and response bias (O_hit rate_/O_false alarm rate_). For consistency and clarity, we used the term "discrimination index" to represent the d-prime calculated for the auditory detection task (d-prime = Z_hit_ – Z_false alarm_). Response bias indicates whether participants are using a more stringent (conservative) or more lenient (liberal) criterion for making judgments, and is calculated based on the hit rate and false alarm rate. Then, we conducted the Pearson correlation analyses to examine the relationships between the WM performance obtained from the verbal and spatial WM span tasks, as well as the dual 2-back task, and the auditory detection performance measures. To address the multiple comparisons inherent in these analyses, we adjusted the significance level to 0.004 (i.e., 0.05/12) using the Bonferroni correction for the 12 correlations tested. Finally, for those variables showing significant correlations, we performed both linear and quadratic regression analyses to determine whether the relationship between WM performance and auditory detection performance follows a linear pattern or a U-shaped/inverted U-shaped curve. For those variables where the correlations were not significant, we conducted quadratic regression analyses to examine whether the relationship between WM performance and auditory detection performance follows a U-shaped/inverted U-shaped curve.

### Results

#### Relationship between WM and auditory detection performance

The Pearson correlation analyses indicated that dual 2-back task performance was significantly correlated with several auditory detection measures, including hit rate (*r* = 0.449, *p* < 0.004), false alarm rate (*r* = −0.456,* p* < 0.004), discrimination index (*r* = 0.625,* p* < 0.004). However, response bias (*r* = 0.289, *p* = 0.008) was not significant after correction for multiple comparisons (*p* > 0.004) (Table 1). Other correlations between the WM performance measures and auditory detection performance were not significant (all *p* > 0.004) (Table 1).

**Table 1:** Pearson Correlation Coefficients between different types of WM and Auditory Detection Performance

WM performance	Auditory detection performance			
	Hit rate	False alarm rate	Discrimination index	Response bias
VWM Span	0.156	−0.166	0.222	−0.002
SWM Span	0.174	−0.121	0.175	0.050
Dual 2-back	0.449*	−0.456*	0.625*	0.289

“*” denotes significance at *p* < 0.004 after Bonferroni correction. VWM refers to the verbal working memory; SWM refers to the spatial working memory

#### Aeronautical decision-making performance

Participants exhibited an average accuracy of 83.30% (*SD* = 6.11%) in the aeronautical decision-making task, with a mean reaction time of 1712 ms (*SD* = 329 ms). To examine potential trade-offs with auditory detection task performance, we calculated correlations between key metrics: accuracy and response time for the aeronautical decision-making task, and hit rate and discrimination index for the auditory detection task. The accuracy of the aeronautical decision-making task was not significantly correlated with the discrimination index (*r* = 0.049, *p* > 0.05) or hit rate (*r* = −0.012,* p* > 0.05) of the auditory alarm detection task. Similarly, response time in the aeronautical decision-making task was not significantly correlated with the discrimination index (*r* = −0.106, *p* > 0.05) or hit rate (*r* = −0.043, *p* > 0.05) of the auditory alarm detection task. These results indicated that participants were able to accurately understand and execute the aeronautical decision-making task, and this task did not negatively impact their performance on the auditory alarm detection task.

#### Regression results

##### Verbal working memory (VWM) capacity and auditory task performance

For the discrimination index, the quadratic regression analysis, including both VWM span score and its square term as predictors, showed that the quadratic model was significant (*F* (2, 81) = 4.403, *p* < 0.05, *R*^*2*^ = 0.098), indicating that 9.8% of the variance in discrimination index is explained by VWM performance. The quadratic term significantly predicted the discrimination index (*β* = −6.390, *t* = −2.089, *p* < 0.05). Similarly, for the hit rate, the quadratic regression analysis showed that the quadratic model was significant (*F* (2, 81) = 3.370, *p* < 0.05, *R*^*2*^ = 0.077). The linear term (VWM span score) had a coefficient of 2.047 (*t* = 2.339, *p* < 0.05), indicating a significant positive relationship with the discrimination index. The quadratic term (VWM span score squared) had a coefficient of -1.297 (*t* = − 2.143, *p* < 0.05), indicating a significant negative relationship. These results suggest that the relationship between VWM span score and both discrimination index and hit rate is curvilinear.

For the false alarm rate and response bias, the quadratic regression analysis showed that the quadratic regression model was not significant (false alarm rate: *F* (2, 81) = 1.176, *p* = 0.314, *R*^*2*^ = 0.028; response bias: *F* (2, 81) = 1.021, *p* = 0.365, *R*^*2*^ = 0.025).

##### Spatial working memory (SWM) capacity and auditory task performance

For the discrimination index and hit rate, the quadratic regression analysis, including both SWM span score and its square term as predictors, showed that the quadratic regression model was significant (discriminate index: *F* (2, 81) = 3.629, *p* < 0.05, *R*^*2*^ = 0.082; hit rate: *F* (2, 81) = 4.244, *p* < 0.05, *R*^*2*^ = 0.095). The linear term (SWM span score) had a coefficient of 32.578 (*t* = 2.241, *p* < 0.05), indicating a significant positive relationship with the discrimination index. The quadratic term (SWM span score squared) had a coefficient of −20.113 (*t* = − 2.132, *p* < 0.05), indicating a significant negative relationship. Similarly, for the hit rate, the linear term had a coefficient of 6.444 (*t* = 2.512, *p* < 0.05), and the quadratic term had a coefficient of −4.001 (*t* = −2.404, *p* < 0.05), indicating significant effects. These results suggest that the relationship between SWM span score and both discrimination index and hit rate is curvilinear.

For the false alarm rate and response bias, the quadratic regression analysis showed that the quadratic regression model was not significant (false alarm rate: *F* (2, 81) = 1.601, *p* = 0.208, *R*^*2*^ = 0.038; response bias: *F* (2, 81) = 0.175, *p* = 0.840, *R*^*2*^ = 0.004).

##### Executive function of WM and auditory task performance

For the discrimination index, the linear regression results showed that the regression model was significant, *F* (1, 82) = 52.434, *p* < 0.01, with an *R*^*2*^ = 0.390, indicating that 39% of the variance in discrimination index is explained by dual 2-back performance. Dual 2-back performance significantly predicts the discrimination index (*β* = 1.152, *t* = 7.241, *p* < 0.01), supporting a linear relationship. However, the quadratic regression analysis, including both dual 2-back performance and its square term as predictors, showed that the quadratic term did not significantly predict the discrimination index (*β* = −0.114, *t* = −0.526, *p* = 0.60), suggesting no evidence for an inverted U-shape association.

Similarly, for the hit rate and false alarm rate, the linear regression model were significant (hit rate: *F* (1, 82) = 20.715, *p* < 0.01, *R*^*2*^ = 0.202; alarm rate: *F* (1, 82) = 21.472, *p* < 0.01, *R*^*2*^ = 0.208), indicating that dual 2-back performance significantly predicted both measures (hit rate: *β* = 0.148, *t* = 4.551, *p* < 0.01; false alarm rate: *β* = −0.074, *t* = −4.634, *p* < 0.01), supporting a linear relationship. However, the quadratic regression model for the hit rate did not show a significant prediction by the square term of dual 2-back performance (*β* = −0.025, *t* = −0.568, *p* = 0.572) nor did it for the false alarm rate (*β* = 0.027, *t* = 1.262, *p* = 0.211), suggesting no evidence for an inverted U-shaped or U-shaped association.

Given the non-significant Pearson correlation between dual 2-back task performance and response bias, we conducted a quadratic regression analysis. The results showed that neither the dual 2-back performance nor its square term significantly predicted the response bias (*β* = 1.431, *t* = 1.251, *p* = 0.215; *β* = -0.227, *t* = −0.561,* p* = 0.577, respectively).

## Discussion

This experiment reveals significant quadratic regression models for both the discrimination index and hit rate in relation to VWM and SWM capacities, indicating curvilinear relationships. This finding suggests that there appears to be an optimal range of WM capacity where auditory task performance is enhanced. As the VWM or SWM span scores initially increase, the discrimination index and hit rate improve, which might be attributed to better storage and processing capabilities facilitating the auditory detection task. However, beyond a certain point, further increases in these span scores lead to a decline in performance, potentially due to factors like excessive cognitive load or interference that arise with very high WM capacities. These patterns were not observed in the analysis of false alarms and response bias. Previous studies also indicated that cognitive load or WM load did not lead to significant changes in response bias (Raveh & Lavie, 2015; Zhu et al., 2023). Response bias, which reflects decision-making strategies, may be more influenced by various factors beyond WM capacity (Bedi et al., 2024). Moreover, it is imperative to acknowledge the limitations inherent to our models, as evidenced by the relatively modest *R*^2^ values obtained from the quadratic regression analyses. These findings suggest that the relationship between the storage function of WM and auditory alarm deafness does not entirely align with the dual-route hypothesis (Hannon & Richards, 2010). Future research should explore additional cognitive and contextual factors that might affect response criteria and performance in auditory alarm detection tasks, and refine models to better capture the complexity of these relationships.

Unlike the storage function of WM, the results pertaining to the executive function of WM revealed a linear relationship, consistent with the predictions of a single-route model. Specifically, our regression analysis indicated a significant association between executive function of WM and auditory alarm detection performance. Individuals with higher executive function of WM were more likely to detect the critical auditory alarm in the high-load tasks than individuals with lower executive function of WM. Prior studies have found no association between the executive function of WM and auditory alarm deafness (Dehais et al., 2019; Kreitz et al., 2016), although this may be related to the single visual n-back task used in those studies. However, the dual n-back task we chose requires individuals to simultaneously pay attention to information in both the visual and auditory modalities, and to distinguish the target stimuli from the non-target stimuli; this involves information update, inhibition of non-target stimuli, and the switching of audiovisual information (Strobach et al., 2014). Accordingly, it may be that individuals with high performance in the dual n-back task have higher efficiency in encoding and processing information and thus can coordinate their cognitive resources between target processing and non-target inhibition relatively well. Moreover, the cognitive components shared between WM tasks and other cognitive skills affect the predictive relationship between them (Soveri et al., 2017; Waris et al., 2015). Therefore, the dual n-back task may be more closely related to cross-modality auditory alarm response sensitivity than the single-modality n-back task.

These results from experiment 1 demonstrate that the storage and executive functions of WM played different roles in auditory alarm detection ability. Individuals with high WM executive function were less likely to exhibit auditory alarm deafness in the high-load context. Hence, the question of whether enhancement of the executive function of WM can improve auditory alarm response sensitivity under high-load tasks needs to be further explored. Experiment 2 was designed to address this issue.

## Experiment 2

### Materials and methods

#### Participants

We conducted a power-test in G*Power version 3.1 software (Faul et al., 2009). With effect size (d) 0.167 or 0.175 (the effect size for the discrimination index or training level difference among tDCS combined with training group, sham tDCS combined with training group and tDCS-only group in Martin et al. (2013)), the type of error (α) to 0.05, the statistical test power (1-β) to 0.95, we required at least 75 or 69 participants. The training sample size used in this experiment exceeds the estimated sample size mentioned above.

Ninety (50 female, 40 male) undergraduates and postgraduates from Shaanxi Normal University participated in this experiment, ranging in age from 17 to 28 (mean age 19.57, *SD* = 2.32). Participants were randomly assigned to three different groups, namely the anodal stimulation combined with training group (17 female, 13 male), the sham simulation combined with training group (17 female, 13 male) and the tDCS alone group (16 female, 14 male). The gender difference between different groups is not significant (*χ*^*2*^(2) = 0.09, *p* = 0.956); the age difference is not significant (*F* (2.87) = 1.198, *p* = 0.307). All participants had normal or corrected-to-normal vision, normal hearing, no history of nerve damage or mental illness, no history of epilepsy, no drug or alcohol dependence, and no metal implants in the skull. None of the participants had participated in a similar experiment and they were naïve to the purpose of the experiment. This experiment was approved by the Academic Ethics Committee of Shaanxi Normal University for human participants research (approval number: HR2020-10-001). Participants signed an informed consent form before the experiment, and each participant received remuneration upon completion of the experiment.

#### Procedure

**Fig. 3:**
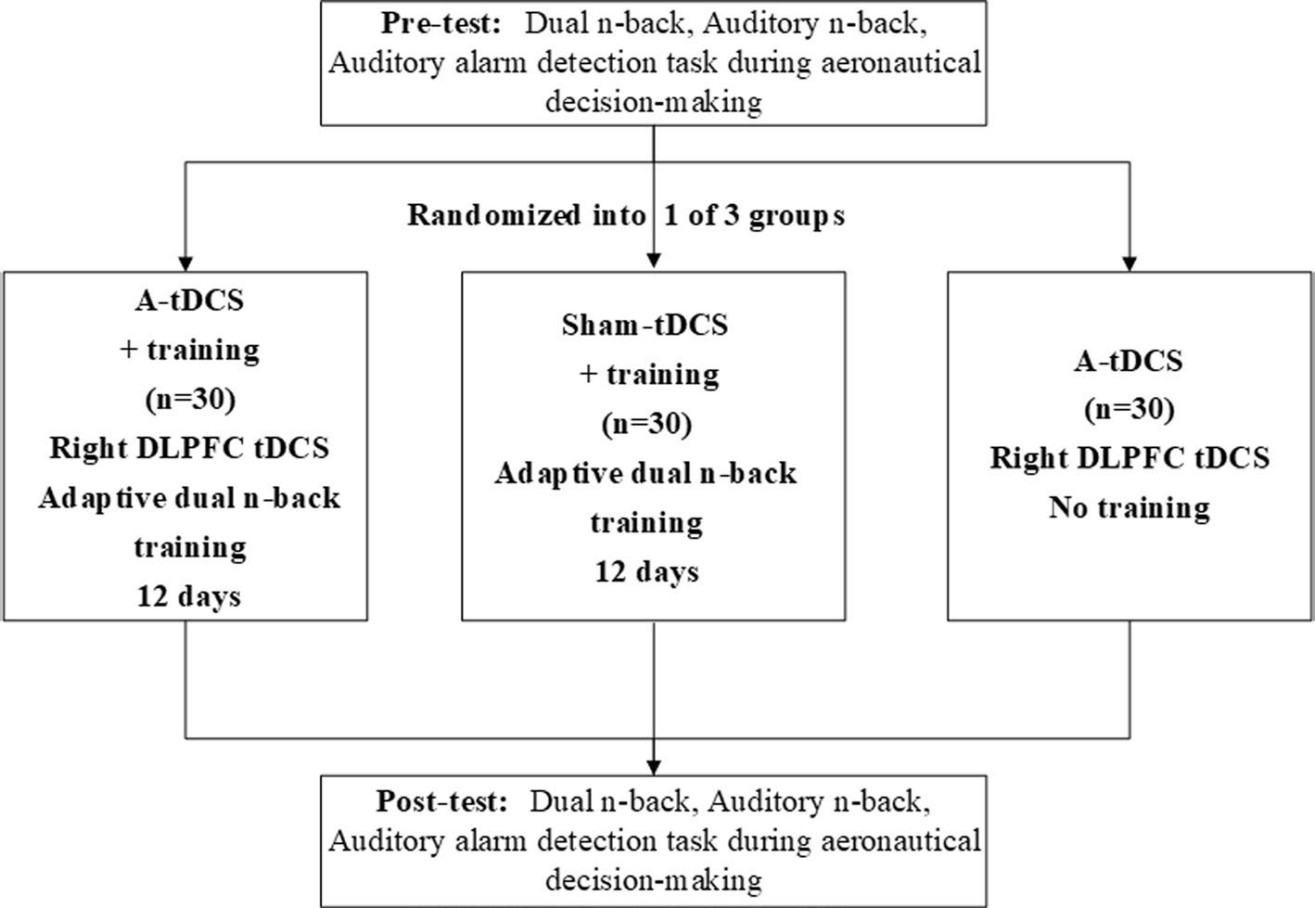
Study design and progress of participants through trial

A pre-test-intervention-post-test design was adopted in this experiment. The pre-test and post-test tasks included an audio-visual dual n-back task, an auditory n-back task, and auditory alarm detection task during aeronautical decision-making. To increase the difficulty and relevance, we selected the visual letter-auditory pitch dual n-back task, as it better aligns with the auditory pitch variations relevant to the alarm stimuli. An adaptive dual n-back training task was chosen due to the fact that such tests have demonstrated transfer effects in healthy participants (Jaeggi et al., 2008, 2010). All auditory stimuli were processed using Adobe Audition CC 2019 software, with a sampling size of 16 bits and 44 kHz. The flowchart of the entire experiment is shown in Fig. 3.

We employed a single-blind experimental design, where participants were unaware of the type of tDCS they were receiving, while the experimenter was aware of the tDCS condition assigned to each participant. Prior to the baseline assessment, participants were randomly assigned to one of three experimental groups: anodal tDCS with training, sham tDCS with training, and the tDCS alone group. Before beginning the training, all participants were asked to perform the pre-test task. The anodal tDCS with training group received 20 min of real current stimulation each time and then completed an adaptive dual n-back training exercise. To ensure that the sham tDCS with training group experienced an itching feeling similar to that of real stimulation, the sham tDCS stimulus comprised 30 s of 1.5 mA anodal tDCS, with 15 s ramp up and down. The tDCS alone group did not perform an adaptive dual n-back training, and only performed a dual n-back task after 20 min of real current stimulation. Each time the tDCS device was used, the participants were asked whether they felt unwell and whether they intended to continue the experiment. The training lasted for 12 working days, and the post-test task was completed after the training.

#### Training task

**Fig. 4:**
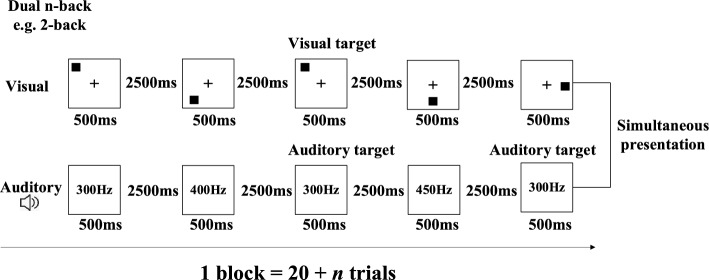
The dual n-back task that was used as the training task, illustrated for a 2-back condition

The adaptive dual n-back training task was conducted on a 21-inch desktop computer using MATLAB (R2014b) (MathWorks Inc., Natick, MA) and the Psychophysics Toolbox (version 3.0.15) (Brainard, 1997). Two sets of stimuli were presented to participants simultaneously at a rate of 3 s per stimulus (Fig. 4). One set consisted of a sine pure tone with varying pitches, while the other consisted of individual spatial locations marked with a black square on the screen. Participants were instructed to remember the pitch of the pure tone that was presented sequentially through the headphones and the position of the black square, and then to determine whether the pitch of the pure tone presented matched the one presented *n* (*n* = 1, 2, 3……) items before in the sequence, as well as whether the position of the presented square was the same as the one presented *n* (*n* = 1, 2, 3……) items before in the sequence. The sine pure tone was randomly presented at 200 Hz, 250 Hz, 300 Hz, 350 Hz, 400 Hz, 450 Hz, 500 Hz, or 550 Hz. Participants made responses by pressing the letter “A” of a standard keyboard for the visual target, “L” for the auditory target, and the keys “A” and “L” simultaneously for both the visual and auditory targets. In each training session, the task was adaptive, meaning that if the participant responded at least 90% correctly in both tasks, they advanced to the next level (e.g., from 2-back to 3-back). If the participant responded 70% or less correctly during a run in either of the tasks, they fell to a lower level (e.g., from 3-back to 2-back), with the lowest possible level being 1-back. Otherwise (i.e., accuracy between 70 and 90%), the n-back level remained constant. Participants received feedback on their performance on the visual and auditory n-back tasks after each block and were informed about the n-back level of the next run. Each training session consisted of 20 blocks, with each block including 20 + n trials. There were six visual and auditory targets and two visual-auditory targets per block. The first training started from the 1-back task, and each subsequent training level started from the level of the last block of the previous training.

#### Assessment of pre-test and post-test tasks

*Single auditory n-back* The single n-back tasks were the component (auditory) tasks of the dual n-back task. A sine pure tone was randomly presented at 170 Hz, 220 Hz, 270 Hz, 320 Hz, 370 Hz, 420 Hz, 470 Hz, and 520 Hz. Participants were instructed to remember the pitches of the pure tones that were presented sequentially through the headphones and to determine whether the pitch of the pure tone presented matched the one presented *n* (*n* = 1, 2, 3……) items before in the sequence. Participants made responses by pressing the letter “A” of a standard keyboard for a target, and “L” for a non-target.

*Auditory Alarm Detection Task During Aeronautical Decision-Making* This task is the same as used in Experiment 1, specifically focusing on the high-load condition. The procedure is the same as shown in Fig. 1.

*Dual n-back task* The procedure of this task is the same as was used in the training task (see Fig. 4).

#### tDCS set-up

**Fig. 5:**
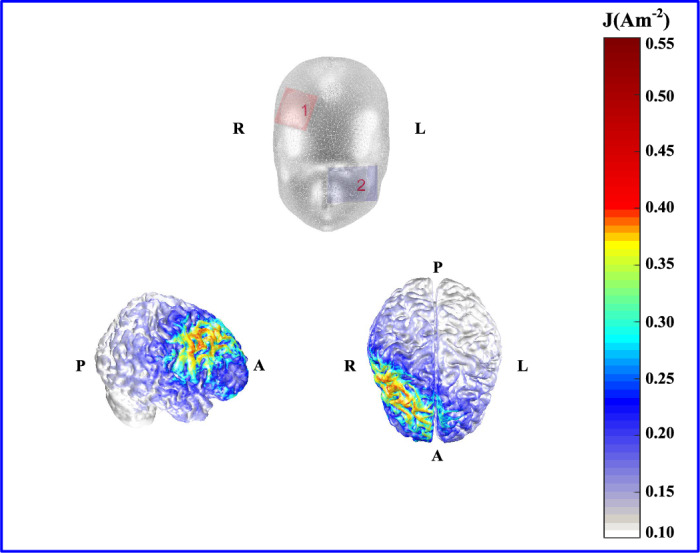
Current density distributions for anodal DLPFC tDCS

The protocols for tDCS were administered using the DC-STIMULATOR PLUS (neuroConn GmbH, Germany) with a pair of rubber electrodes attached to saline-soaked sponges measuring 5 × 7 cm^2^. The anode electrode was placed over the right DLPFC at F4 according to the 10–20 international system for electroencephalograms (EEG), while the cathode electrode was placed on the left cheek (see Fig. 5). A prior study has shown that using an extracephalic position for the reference electrode (cathode) can mitigate confounding effects from inhibitory cathodal effects at other cortical sites (Martin et al., 2013). A moderate intensity of 1.5 mA has been found to effectively enhance WM performance in healthy individuals (Berryhill, 2017; Berryhill et al., 2010). Therefore, a constant electric current of 1.5 mA was delivered for a duration of 20 min, with a fade-in and fade-out time of 15 s each. In order to reduce scalp resistance and scalp pain during stimulation, the sponge on the electrode plate was soaked with normal saline before each stimulation. The simulation of current density distributions generated by tDCS, performed using the Comets toolbox (available at http://www.cometstool.com), showed that this montage covers the right DLPFC area (Fig. 5).

The top panel shows electrode locations on the scalp surface and contralateral cheek. “1” and “2” refer to the right DLPFC and left cheek, respectively. The bottom panels show cortical current density distributions from a frontal view and an overhead view, respectively. “L”, “R”, “A”, and “P” stand for left, right, anterior and posterior, respectively.

### Data analysis

First, the pretest performance of each of the three groups on the dual n-back task, single auditory n-back task, and auditory alarm detection task were calculated to test whether there were significant differences in the baseline levels of performance for each task. For the dual n-back training task, one-way ANOVA analysis was used to determine whether there were significant differences in the training level (n), d-prime, and response time (RT) among the three groups (i.e., anodal tDCS with training, sham tDCS with training, and the tDCS alone group). The d-prime (d-prime = Z_hit_—Z_false alarm_) was calculated using the method of signal detection theory and is commonly used as a measurement index for the accuracy of WM n-back tasks (Haatveit et al., 2010).

Second, to examine the enhancement of WM, the training level (*n*), d-prime, and RT of the dual n-back task were analyzed using three separate 3 × 2 mixed-design ANOVAs, including a between-subjects factor of group (anodal tDCS with training, sham tDCS with training, and tDCS alone group) and within-subject factor of time (pre-test vs. post-test).

Finally, the transfer effect of WM training on the auditory n-back task and auditory alarm detection task were calculated. For the auditory alarm detection task, the hit rate, false alarm rate, discrimination index, and response bias were analyzed using four separate 3 × 2 mixed-design ANOVAs, including a between-subjects factor of group (anodal tDCS with training, sham tDCS with training, and tDCS alone group) and within-subject factor of time (pre-test vs. post-test). For the auditory n-back task, the d-prime and RT were analyzed using two separate 3 × 2 mixed-design ANOVAs, including a between-subjects factor of group (anodal tDCS with training, sham tDCS with training, and tDCS alone group) and within-subject factor of time (pre-test vs. post-test). The significance level for all statistical tests was set at *p* < 0.05. More stringent thresholds of *p* < 0.01 and *p* < 0.001 are also reported where applicable to highlight more robust findings. The specific thresholds used for each analysis are indicated in the results section.

### Results

#### Performance on the WM training task

##### Pre-test results

One-way ANOVA analysis showed that there were no significant differences in the pre-test performance on the dual 2-back task among the three groups (d-prime: *F* (2.87) = 1.877, *p* = 0.159, *η*_*p*_^*2*^ = 0.041, 90%CI [0.000–0.114]; n: *F* (2.87) = 1.155, *p* = 0.320, *η*_*p*_^*2*^ = 0.026, 90%CI [0.000–0.087]; RT: *F* (2.87) = 1.414, *p* = 0.249,* η*_*p*_^*2*^ = 0.032, 90%CI [0.000–0.097]).

##### Enhancement effect

**Fig. 6:**
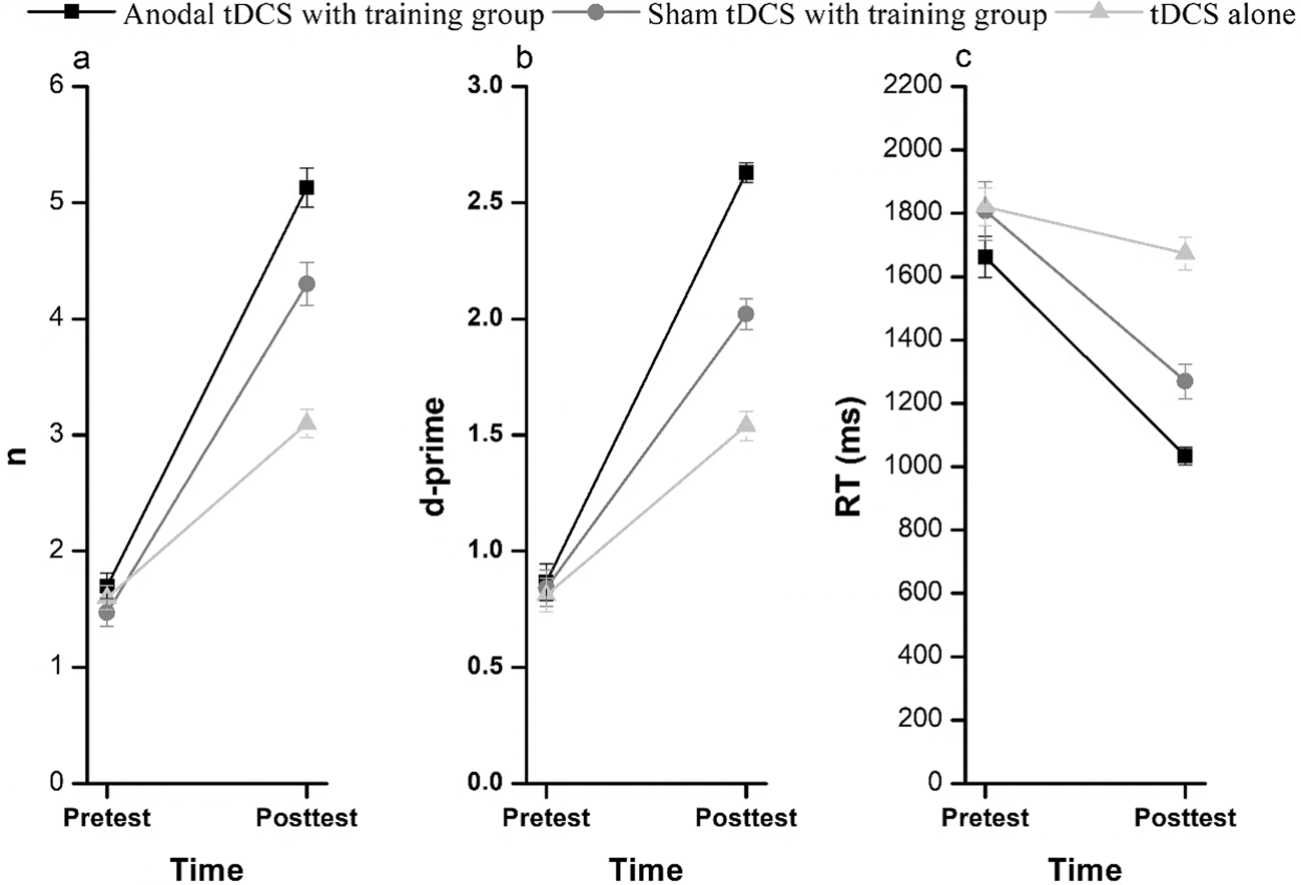
Pre-test and post-test WM performance for three groups. The pre-test and post-test performance of the three groups as measured by training level (**a**), d-prime (**b**) and response time (RT) (**c**)

*Training level (n) and d-prime* For the training level (n), the 3 × 2 mixed-design ANOVA analysis showed that the main effect of groups was significant, *F* (2.87) = 24.374,* p* < 0.01, *η*_*p*_^*2*^ = 0.359, 90%CI [0.219–0.460]. The main effect of time was also significant, *F* (1.87) = 681.255,* p* < 0.01, *η*_*p*_^*2*^ = 0.887, 90%CI [0.850–0.908]. The interaction effect between groups and time was significant, *F* (2.87) = 33.179,* p* < 0.01, *η*_*p*_^*2*^ = 0.433, 90%CI [0.294–0.527]. Simple effects analysis revealed significant differences in post-test performance among the anodal tDCS with training group, the sham tDCS with training group, and tDCS alone group (*ps.* < 0.01). Additionally, the post-test performance was significantly better than the pre-test performance in the anodal tDCS with training group (*p* < 0.01), the sham tDCS with training group (*p* < 0.01), and the tDCS alone group (*p* < 0.01) (Fig. 6a, Table 2).

**Table 2:** The pre-test and post-test performance and gains (post-test – pre-test) of the three groups

Performance	Anodal tDCS with training			Sham tDCS with training			tDCS alone		
	n	d-prime	RT	n	d-prime	RT	n	d-prime	RT
Pre-test	1.70(0.60)	0.87(0.43)	1661(354)	1.47(0.63)	0.84(0.43)	1807(507)	1.60(0.56)	0.81(0.39)	1820(332)
Post-test	5.13(0.94)	2.63(0.23)	1035(150)	4.30(1.02)	2.02(0.37)	1270(302)	3.10(0.66)	1.54(0.35)	1673(287)
Gains	3.43(0.97)	1.76(0.59)	−626(396)	2.83(1.12)	1.18(0.45)	−537(514)	1.50(0.68)	0.73(0.51)	−147(407)

The unit for reaction time (RT) is milliseconds (ms); the numbers within parentheses represent the standard deviation (SD)

For the d-prime, the 3 × 2 mixed-design ANOVA analysis showed that the main effect of groups and time was significant (groups: *F* (2.87) = 34.506,* p* < 0.01, *η*_*p*_^*2*^ = 0.442, 90%CI [0.304–0.535]; time: *F* (1.87) = 498.388,* p* < 0.01, *η*_*p*_^*2*^ = 0.851, 90%CI [0.803–0.880]). The interaction effect between groups and time was also significant, *F* (2.87) = 29.426,* p* < 0.01, *η*_*p*_^*2*^ = 0.404, 90%CI [0.263–0.501]. Simple effects analysis revealed a significant difference in post-test performance among the three groups (*ps*. < 0.01). The post-test performance in all three groups were significantly better than the pre-test performance(*ps*. < 0.01) (Fig. 6b, Table 2).

**Fig. 7:**
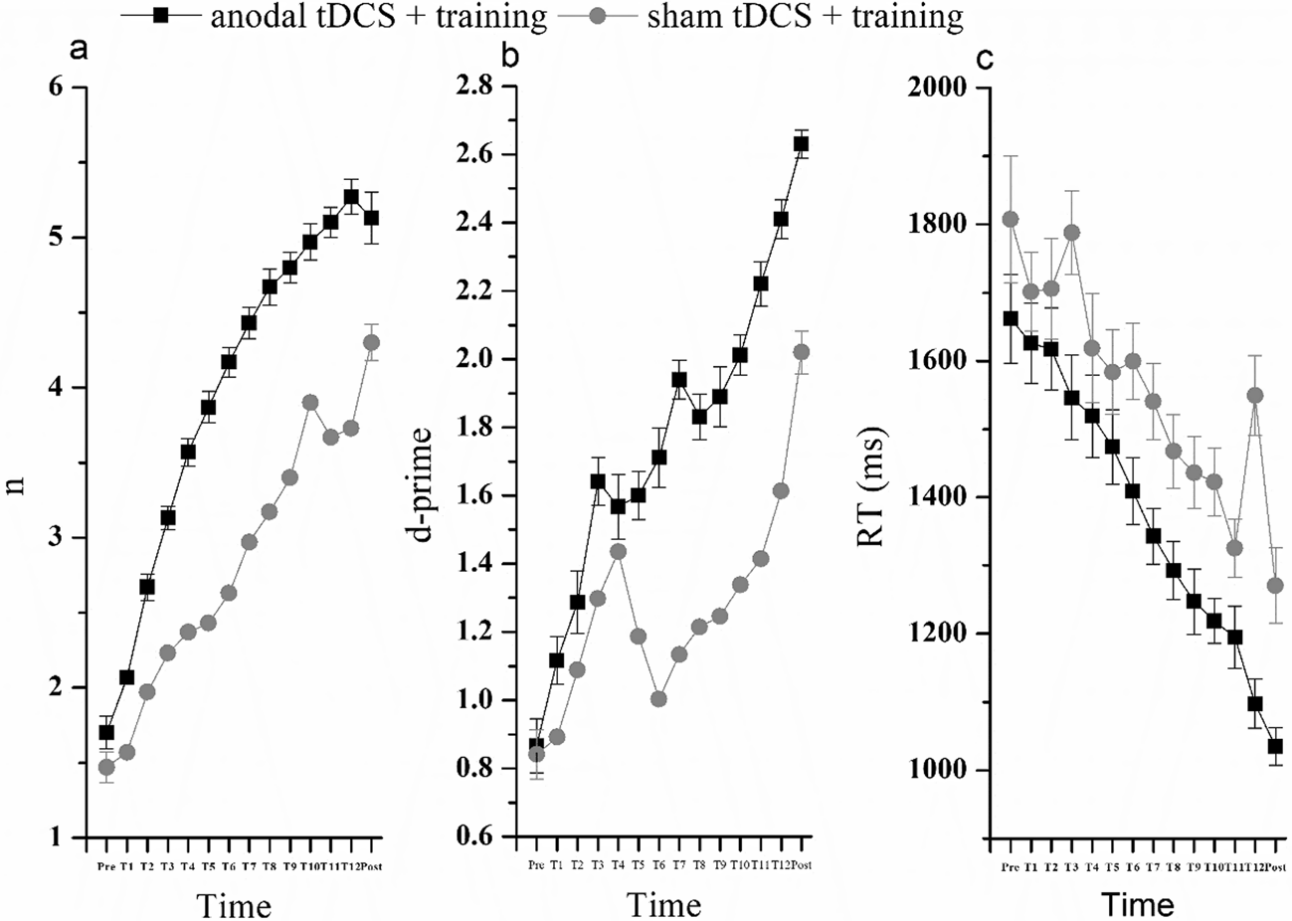
The enhancement effect of anodal tDCS in the training group and sham tDCS with training group. The curves of changes in training level (**a**), d-prime (**b**) and response time (**c**) in these two groups

In addition, we calculated the average training level (n) and d-prime achieved by the participants every day. The curves of changes in the level and discrimination index in the anodal tDCS with training group and the sham tDCS with training group are shown in Fig. 7a and b.

*Response time (RT)* For the RT, the 3 × 2 mixed-design ANOVA analysis showed that the main effect of groups and time was significant (groups: *F* (2.87) = 18.058,* p* < 0.01, *η*_*p*_^*2*^ = 0.293, 90%CI [0.156–0.399]; time: *F* (1.87) = 87.796,* p* < 0.01, *η*_*p*_^*2*^ = 0.502, 90%CI [0.377–0.592]). The interaction effect between groups and time was also significant, *F* (2.87) = 9.996,* p* < 0.01, *η*_*p*_^*2*^ = 0.187, 90%CI [0.068–0.293]. Simple effects analysis showed that there were significant differences in post-test performance among the three groups(*ps*. < 0.01). The post-test performance in the two training groups (i.e., anodal tDCS and sham tDCS training groups) were significantly better than the pre-test performance (*ps*. < 0.01). There was no significant difference between pre-test and post-test performance in the tDCS alone group (*p* > 0.05) (Fig. 6c). The curves of changes in RT for the anodal tDCS with training group and the sham tDCS with training group are shown in Fig. 7c.

#### Performance on non-training tasks

##### The results of auditory alarm detection task

*Pre-test performance* One-way ANOVA analysis showed that there were no significant differences in the pre-test hit rate, false alarm rate, discrimination index, or response bias among the three groups (hit rate: *F* (2.87) = 0.469, *p* = 0.627, *η*_*p*_^*2*^ = 0.011, 90%CI [0.000–0.053]; false alarm rate: *F* (2.87) = 2.871, *p* = 0.062, *η*_*p*_^*2*^ = 0.062, 90%CI [0.000–0.144]; discrimination index: *F* (2.87) = 2.040, *p* = 0.136, *η*_*p*_^*2*^ = 0.045, 90%CI [0.000–0.119]; response bias: *F* (2.87) = 0.064, *p* = 0.938, *η*_*p*_^*2*^ = 0.002, 90%CI [0.000–0.021]).

**Fig. 8:**
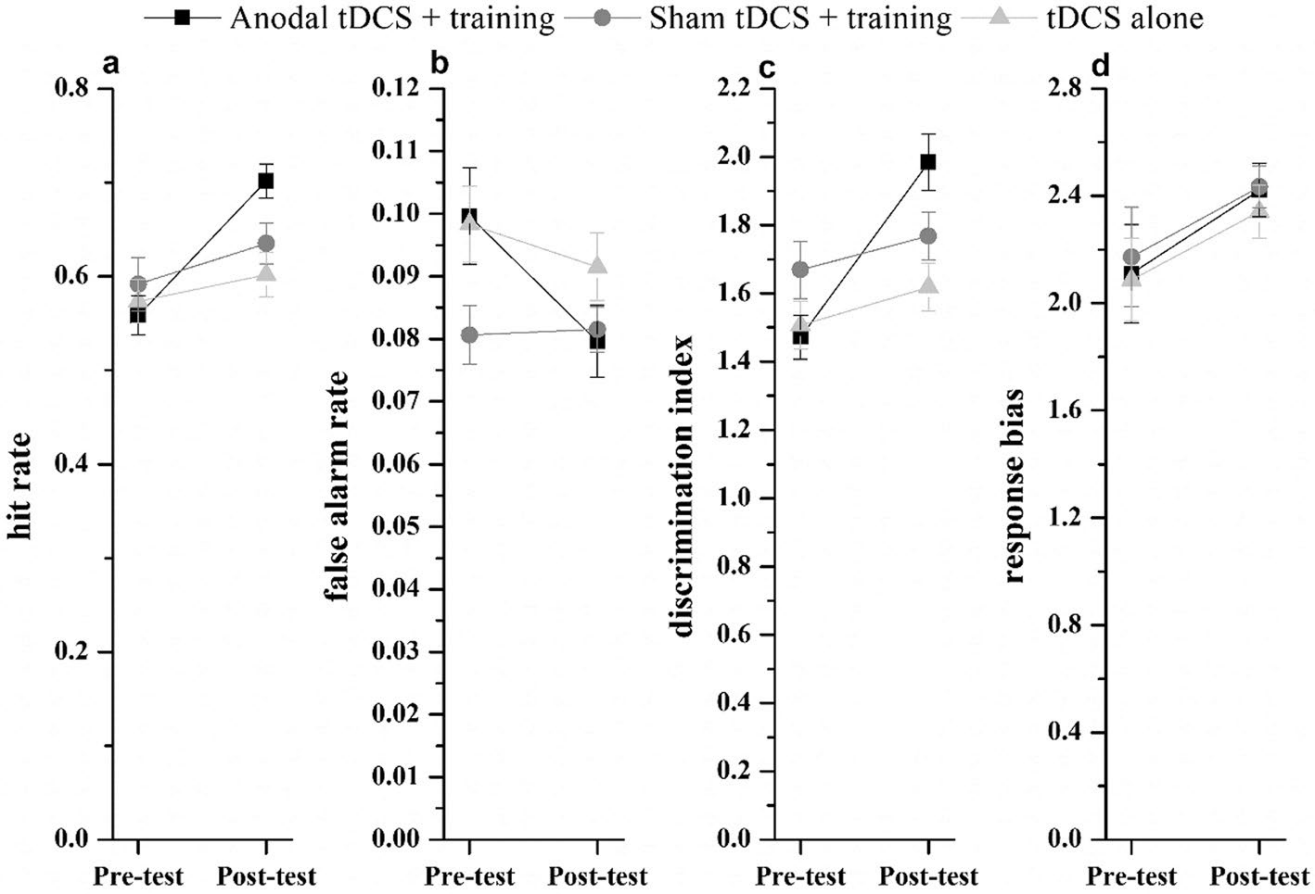
The pre-test and post-test performance of the three groups on the auditory alarm detection task. The pre-test and post-test performance of the three groups as measured by the hit rate (**a**), false alarm rate (**b**), discrimination index (**c**) and response bias (**d**)

*Enhancement effect* For the hit rate and discrimination index, the 3 × 2 mixed-design ANOVA analysis showed that the main effect of time was significant (hit rate: *F* (1.87) = 31.400, *p* < 0.01, *η*_*p*_^*2*^ = 0.265, 90%CI [0.139–0.379]; discrimination index:* F* (1.87) = 39.505, *p* < 0.01, *η*_*p*_^*2*^ = 0.312, 90%CI [0.182–0.424]). The main effect of groups was non-significant (hit rate: *F* (2.87) = 1.140,* p* = 0.325, *η*_*p*_^*2*^ = 0.026, 90%CI [0.000–0.086]; discrimination index:* F* (2.87) = 1.952,* p* = 0.148, *η*_*p*_^*2*^ = 0.043, 90%CI [0.000–0.116]). The interaction effect between time and groups was significant (hit rate: *F* (2.87) = 7.965, *p* < 0.01, *η*_*p*_^*2*^ = 0.155, 90%CI [0.046–0.258]; discrimination index: *F* (2.87) = 12.549,* p* < 0.01, *η*_*p*_^*2*^ = 0.224, 90%CI [0.097–0.331]). Simple effects analysis showed that the hit rate and discrimination index in the post-test were significantly higher than the pre-test for the anodal tDCS with training group, respectively (*ps.* < 0.01), whereas no significant difference was observed between the pre-test and post-test in the hit rate and discrimination for the sham tDCS with training group and the tDCS alone group (*ps*. > 0.05) (Fig. 8a, c).

For the false alarm rate, the main effect of time was significant, *F* (1.87) = 14.006, *p* < 0.01, *η*_*p*_^*2*^ = 0.139, 90%CI [0.044–0.249]. The main effect of groups was non-significant, *F* (2.87) = 1.734,* p* = 0.183, *η*_*p*_^*2*^ = 0.038, 90%CI [0.000–0.109]. The interaction effect between time and groups was significant, *F* (2.87) = 6.994,* p* < 0.01, *η*_*p*_^*2*^ = 0.139, 90%CI [0.036–0.239]. Simple effects analysis showed that the false alarm rate in the post-test was significantly lower than the pre-test for the anodal tDCS with training group (*p* < 0.01). No significant differences between pre-test and post-test performance in the sham tDCS with training group or the tDCS alone group were observed (*ps*. > 0.05) (Fig. 8b).

For the response bias, the main effect of time was significant, *F* (1.87) = 8.505, *p* < 0.01, *η*_*p*_^*2*^ = 0.089, 90%CI [0.017–0.192]. The main effect of groups was non-significant, *F* (2.87) = 0.036,* p* = 0.965, *η*_*p*_^*2*^ = 0.001, 90%CI [0.000–0.016]. The interaction effect between time and groups was also non-significant, *F* (2.87) = 0.155,* p* = 0.857, *η*_*p*_^*2*^ = 0.004, 90%CI [0.000–0.025] (Fig. 8d).

### The results of the auditory n-back task

One-way ANOVA analysis showed that there were no significant differences in either the pre-test d-prime or pre-test RT among the three groups (d-prime: *F* (2.87) = 0.708, *p* = 0.495, *η*_*p*_^*2*^ = 0.016, 90%CI [0.000–0.067]; RT: *F* (2.87) = 2.372, *p* = 0.099, *η*_*p*_^*2*^ = 0.052, 90%CI [0.000–0.130]).

For the d-prime index, the 3 × 2 mixed-design ANOVA analysis showed that the main effect of groups and time was significant (groups: *F* (2.87) = 11.293, *p* < 0.001, *η*_*p*_^*2*^ = 0.206, 90%CI [0.083–0.313]; time: *F* (1.87) = 551.118, *p* < 0.001, *η*_*p*_^*2*^ = 0.864, 90%CI [0.819–0.890]). The interaction effect between groups and time was also significant, *F* (1.87) = 24.318, *p* < 0.001, *η*_*p*_^*2*^ = 0.359, 90%CI [0.218–0.460]. Simple effects analysis showed that there were significant differences between pre-test and the post-test performance in all three groups (*ps*. < 0.01). These results indicate that accuracy (d-prime) on the auditory 2-back task was improved via all three modes of intervention.

For the RT, results showed that the main effect of time was significant, *F* (1.87) = 20.609, *p* < 0.001, *η*_*p*_^*2*^ = 0.192, 90%CI [0.080–0.306]. The main effect of group was non-significant, *F* (2.87) = 1.075, *p* = 0.346, *η*_*p*_^*2*^ = 0.024, 90%CI [0.000–0.084]. The interaction effect between time and group was significant, *F* (2.87) = 3.180, *p* < 0.05, *η*_*p*_^*2*^ = 0.068, 90%CI [0.001–0.153]. Simple effects analysis showed that there were significant differences between the pre-test and the post-test performance in both training groups (*ps*. < 0.01). There was a non-significant difference between pre-test and post-test performance in the tDCS alone group (*p* = 0.572).

## Discussion

This experiment found that anodal tDCS combined with training can not only improve the executive function of WM but also transfer the improvement effect to auditory WM and auditory alarm detection ability. Sham tDCS combined with training was found to improve both the executive function of WM and auditory WM, but did not enhance auditory alarm detection ability. tDCS alone was found to increase the accuracy of task performance on the executive function of WM and auditory WM tasks, but not the response time for both WM tasks and for performing the auditory alarm detection task. These results suggest that anodal tDCS combined with WM training can facilitate a greater enhancement effect and transfer effect than either sham tDCS combined with training or tDCS alone.

Prior studies have shown that anodal tDCS combined with training can generate greater transfer effects for non-trained tasks than either single training or tDCS alone (Au et al., 2016; Ke et al., 2019; Stephens & Berryhill, 2016). The enhanced efficacy of this combined approach in improving auditory alarm response sensitivity in the present study may stem from several underlying mechanisms. Callan et al. (2023) reported that during the concurrent execution of a high-load virtual skateboarding task, increased gamma-band activity in the superior frontal gyrus and DLPFC was observed in the condition where participants correctly identified auditory stimuli (hits) compared to when they missed these stimuli. The findings suggest that the DLPFC play a crucial role in managing dual-task performance by coordinating attentional resources and enhancing task-related processing. The 20-min stimulation protocol applied to the DLPFC should increase the neuronal excitability within this cortical region, thereby enhancing cortical processing efficiency (Nitsche & Paulus, 2000). Moreover, tDCS combined with WM training can improve the processing efficiency of cortical areas and the synchronous oscillations of neurons in the brain regions associated with WM (Jones et al., 2017). We propose that the cumulative effect of multi-session tDCS, as observed in studies by (Monte-Silva et al., 2013), may induce long-term potentiation-like enhancements in cortical excitability and plasticity of the glutamate system, facilitating the processing of input information and establishing new neuronal connection pathways. Consequently, these neuronal changes could in turn enhance WM ability and auditory alarm response sensitivity during high-load tasks. However, the response bias of auditory alarm detection task did not improve through tDCS combined WM training. This finding is consistent with Experiment 1, which also found no relationship between WM and response bias. Since response bias reflects an individual's subjective judgment criterion and can be influenced by factors like personal motivation, future research could use fMRI to more deeply investigate the neural mechanisms underlying response bias.

## General discussion

This study explored the effect of WM and tDCS combined with WM training on auditory alarm detection ability during a high-load aeronautical decision-making task. The results indicated that the relationship between different storage functions of WM and auditory alarm deafness was not strictly linear but rather curvilinear, particularly for discrimination index and hit rate, while no significant relationship was observed for false alarm rate and response bias. However, individual differences in WM executive function are associated with auditory alarm deafness, and tDCS combined with WM training was found to improve auditory alarm response sensitivity during the high-load task. These results show that there is a dissociation between the roles of different functions (i.e., the storage and executive functions) of WM in auditory alarm detection ability, with the relationship between executive function and auditory alarm detection aligning with the single-route theory.

Many studies have shown that the predictive effect of WM on other high cognitive functions mainly comes from its central executive function rather than its storage function (Conway et al., 2002; Engle, 2010; Jung & Haier, 2007). The central executive system of WM, mediated by anterior brain regions, is mainly responsible for attention control and the regulation of cognitive resources (Baddeley, 2010; Constantinidis & Klingberg, 2016; Engle, 2002). In contrast, the storage function of WM, primarily associated with posterior brain regions and involved in the retention and retrieval of information, plays a lesser role in the proactive resource allocation (Arciniega et al., 2018; Olson & Berryhill, 2009). Therefore, in high-load auditory alarm detection tasks, individuals with stronger central executive abilities of WM can better coordinate and optimize attention resources, effectively monitoring and handling unexpected auditory stimuli.

WM training involving a dual-task component is more likely to generate a greater training effect and transfer effect than single-modality training (Klingberg, 2010). The complexity and diversity of training tasks, such as visual-auditory task training, can expand the scope of cognitive transfer (Stevens et al., 2016). The adaptive dual n-back training task used in the current study shares similarities with the auditory alarm detection task under high load, in that both involve dual-task coordination and attentional resource allocation ability in the audiovisual modality under different load conditions. Moreover, the DLPFC not only plays an important role in the executive function of WM (Friedman & Robbins, 2022), but also in the integration and coordination of information from multiple modalities (Miller & Cohen, 2001). It can be speculated that tDCS combined with training may improve attention coordination and information processing ability on cross-modality dual-tasks under different load conditions. The observed improvements in hit rate and discriminative index for the auditory alarm detection task in the tDCS combined with training group provide evidence for this hypothesis.

In both experiments, the response bias in the auditory alarm detection task did not show any significant changes. In Experiment 1, there was no significant linear correlation between different types of WM and response bias, and in experiment 2, neither tDCS combined with training, sham tDCS combined with training, nor tDCS alone led to statistically significant changes in response bias. These findings support earlier studies showing that load effects on inattentional deafness affect only the discriminative index, with no impact on response bias (Macdonald & Lavie, 2011; Raveh & Lavie, 2015). Since sensitivity and response criterion measures are independent in signal detection theory, the lack of effect on response bias does not undermine our main findings regarding changes in response sensitivity. However, Fig. 8d shows that the response bias values tend to increase across all three groups, suggesting that participants may have adjusted their response criteria from pretest to posttest. Specifically, in the tDCS combined with training group, participants initially adopted a more lenient criterion, as evidenced by a higher false alarm rate in the pretest. Over the course of the intervention, this group demonstrated a significant shift toward a stricter criterion, resulting in a notably lower false alarm rate in the posttest.

Prior studies have shown that sufficient training duration can improve the possibility of the far-transfer effect (Jaeggi et al., 2008; von Bastian & Oberauer, 2014). However, in the current study, the sham tDCS combined with 12 days of training improved only the WM itself, with no observable transfer of benefits to the auditory alarm detection task. The enhancement effect of WM in the sham tDCS combined with training group was less pronounced relative to the anodal tDCS combined with training group. These results indicate that tDCS may have potential for further enhancing the WM training effect and its transfer effects. Interestingly, in the sham tDCS combined with training group, we observed a notable increase in RT on day 12 (see Fig. 7c), and the d-prime values exhibited an unusual pattern: an increase on day 4, followed by a sharp decline, and then a subsequent rise (see Fig. 7b). This increase in RT on day 12, coupled with a subsequent decrease during the post-test phase, may reflect potential fatigue or cognitive overload toward the end of the training period, with a possible recovery in performance efficiency after the training concluded. Additionally, the irregular d-prime pattern could indicate fluctuations in participants' sensitivity to the task, potentially influenced by external factors such as variations in participants' motivation and the relocation of the experimental environment due to lab changes. Previous studies have indicated that various factors, including motivation and training duration, can impact the effectiveness of WM training (Berryhill, 2017; von Bastian & Oberauer, 2014). But our study did not systematically monitor these variables throughout the training period, which constitutes a notable limitation. Future research should address this limitation by incorporating measures to track and control for participants' fatigue, motivation, and environmental conditions to better understand their effects on training outcomes and improve the generalizability of findings.

WM plays a crucial role in navigating complex and dynamic working environments, such as those encountered by pilots. In fact, pilots’ situational awareness judgments (Gutzwiller & Clegg, 2012), flight skills (Causse et al., 2020), and reaction times to consecutive automation failures (Jipp, 2016) can be greatly influenced by their WM abilities. tDCS combined with WM training has been found to improve flight simulator skills in an earlier study (Choe et al., 2016) and was found to improve auditory alarm response sensitivity in the current study. These findings suggest that brain enhancement technology combined with behavioral training might offer potential benefits for improving cognitive skills relevant to aviation tasks. However, it is important to acknowledge that this study involved non-pilot participants in a controlled laboratory setting, which may limit the generalizability of the findings to real-world flying situations and professional pilots. Future research, possibly involving trained pilots in real-world aviation settings, is needed to substantiate the practical implications of our findings and explore their potential for reducing human errors and enhancing aviation safety.

## Conclusion

This study explored the influence of WM on auditory alarm detection sensitivity and sought to identify ways to prevent auditory alarm deafness in a simulated high-load aeronautical decision-making scenario. The results indicated that individuals with high WM executive function were more likely to detect the critical auditory alarm than those with low executive function. Stimulation of the right DLPFC, combined with computerized WM training, improved WM and auditory alarm sensitivity during the high-load aeronautical decision-making scenario. These findings suggest that brain enhancement technology and cognitive training can be an effective way to improve WM and auditory alarm sensitivity under high-load contexts.

## Data Availability

The datasets used and/or analyses are available from the corresponding author on reasonable request.
